# Isolation of Human Polymorphonuclear Leukocytes (Granulocytes) from a Leukocyte-Rich Fraction

**DOI:** 10.1100/tsw.2002.831

**Published:** 2002-05-21

**Authors:** John Graham

**Affiliations:** School of Biomolecular Sciences, Liverpool John Moores University, UK

**Keywords:** granulocytes, polymorphonuclear leukocytes, leukocyte-rich plasma, OptiPrep™, iodixanol, density barrier

## Abstract

Human peripheral blood polymorphonuclear leukocytes (PMNs) or granulocytes from a leukocyte-rich plasma (LRP) are banded at an interface between two layers of iodixanol. If the denser layer of iodixanol is omitted the PMNs may alternatively be pelleted. The procedure can be adapted to blood from other species by small changes to the density of the two iodixanol layers. The method works optimally with EDTA- or citrate-anticoagulated blood.

**METHOD TYPE:** extraction, isolation, purification and separation

**SUB METHOD TYPE:** centrifugation

## INTRODUCTION

The polymorphonuclear leukocyte (PMN) fraction from human peripheral blood comprises principally neutrophils with relatively small numbers of basophils and eosinophils. They have densities predominantly above 1.077 g/ml, with the exception of part of the basophil population, while mononuclear cells (lymphocytes + monocytes) have densities below this value (see Fig. 1). Since the density of erythrocytes significantly overlaps that of neutrophils, it is only possible to use a simple OptiPrep™ discontinuous gradient to resolve the mononuclear cells and PMNs from

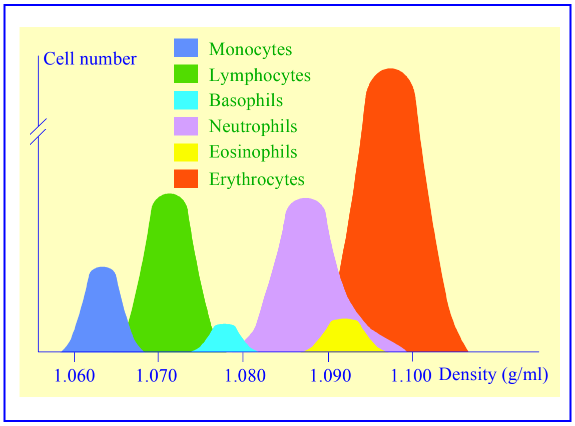

**FIGURE 1.** Density of human blood cells.

a leukocyte-rich plasma (LRP). Isolation of PMNs from whole human blood can only be carried out on Polymorphprep™.

Layering LRP over a barrier whose density is 1.077 g/ml, will allow the PMNs to pellet while the mononuclear cells are retained at the interface. However, to avoid pelleting the PMNs, which could lead to aggregation, a dense cushion (1.090–1.095 g/ml) may be placed beneath the 1.077 g/ml layer in order to band the PMNs.

Subsequent to purification, erythrocyte contamination of the PMN fraction can be eliminated by selective osmotic shock either in cold water or in isotonic NH4Cl (see below).

## MATERIALS AND EQUIPMENT

OptiPrep™ (shake gently before use)
Diluent: 0.85% (w/v) NaCl, 1 mM EDTA, 20 mM HEPES-NaOH, pH 7.4
Dextran: 6% (w/v) dextran ($M_r = 400\text{-}500 \times 10^3$) in 0.9% (w/v) NaCl (optional)
Lysis Buffer (LB): 0.83% (w/v) $NH_4Cl$, 10 mM HEPES-NaOH, pH 7.0 (optional).
1.8 (w/v) NaCl, 20 mM HEPES-NaOH, pH 7.4 (optional)

Plastic conical centrifuge tubes (12–15 ml)
Syringe with metal cannula for underlayering
Plastic Pasteur pipettes
Low-speed (temperature-controlled) centrifuge with swinging-bucket rotor

## METHOD

1. Centrifuge freshly drawn blood in either 1 mM EDTA or 0.38% (w/v) citrate as anticoagulant at $200g_{av}$ for 15–20 min at 18–22°C and harvest the buffy coat in the plasma supernatant from the top of the packed erythrocytes (see Note 1).
2. **OR** add 1 vol of 6% dextran to 9 vol of blood and allow to stand for 20–40 min. Then remove the entire supernatant (see Note 1).
3. Prepare the following density solutions from OptiPrep™ and the Diluent (respectively): 1.077 g/ml, 5 vol + 17 vol and **EITHER** 1.090 g/ml, 8 vol + 22 vol; **OR** 1.095 g/ml, 17 vol + 43 vol (see Notes 2 and 3).

4. Underlayer the 5 ml of LRP with 3–4 ml of 1.077 g/ml solution and the same volume of **EITHER** 1.090 g/ml **OR** 1.095 g/ml.
5. Centrifuge at 18–22°C for 25 min at 800*g*.
6. Using the Pasteur pipette, harvest the two bands in turn; the mononuclear cells from the upper interface and then the PMNs from the lower interface (see Fig. 2).
7. Dilute the PMN suspension with an equal volume of Diluent and collect the cells by centrifugation at 250*g* for 10 min.
8. Resuspend the pellet in a suitable medium (see Note 4).
9. **To remove erythrocyte contamination** from the PMNs, resuspend the cell pellet in 3 ml of LB and incubate at 37°C for 7 min **OR** resuspend the PMNs in 3 ml ice-cold distilled water, then after 30 s add an equal volume of the 1.8% NaCl solution (see Note 4).
10. Harvest by centrifugation and resuspend in a suitable medium.

## NOTES

1. The blood should be processed within 6 h of drawing. Ideally the erythrocytes should be removed as soon as possible after drawing but the subsequent gradient separation of the mononuclear cells and PMNs can be carried out up to 6 h later.
2. If the density of this cushion is 1.090 g/ml, a small percentage of the neutrophils and most of the eosinophils will sediment through this layer. If a density of 1.095 g/ml is chosen, virtually all of the PMNs will be retained by the high-density barrier. On the other hand, fewer of the residual erythrocytes in the LRP will contaminate the PMN band using the lower density cushion.
3. For nonhuman blood, it may be necessary to adjust the density of one or both of the layers. For rat blood for example, increasing the density of the middle layer to 1.080 g/ml may be of benefit (equivalent to approx. 14.2% (w/v) iodixanol). See Ref. [1] for more information on preparing gradient solutions.
4. The cells should be thoroughly, BUT GENTLY, resuspended in the liquid. The RCF used to sediment the cells in step 7 is deliberately chosen to avoid excessive aggregation in the pellet. A satisfactory suspension of this pellet should be achievable with slow aspiration into and expulsion from a Pasteur pipette or gentle agitation.

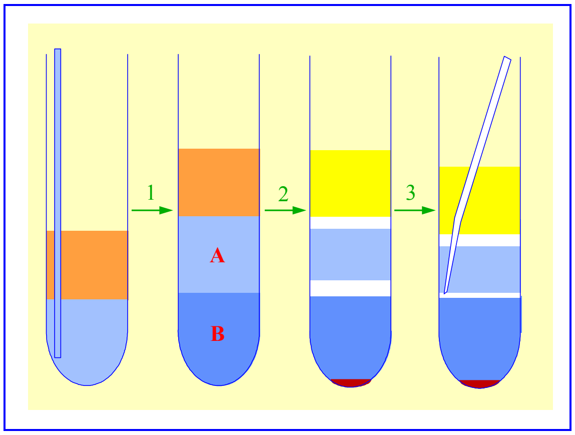

**FIGURE 2**. Separation of PMNs from mononuclear cells from an LRP. (1) LRP layered over A (1.077 g/ml) and B (1.090 or 1.095 g/ml). (2-3) After centrifugation, PMNs harvested from lower interface.

## ACKNOWLEDGEMENTS

The author and TheScientificWorld wish to thank Axis-Shield PoC, AS, Oslo, Norway for their kind permission to adapt OptiPrep™ Application Sheet C11 in the preparation of this Protocol Article.
